# Revised Publication Policies by Higher Education Commission for Health Science Journals

**DOI:** 10.12669/pjms.36.2.2133

**Published:** 2020

**Authors:** Shaukat Ali Jawaid, Masood Jawaid

**Affiliations:** 1 Shaukat Ali Jawaid Chief Editor, Pakistan Journal of Medical Sciences, Karachi - Pakistan; 2 Masood Jawaid Associate Editor, Pakistan Journal of Medical Sciences, Karachi - Pakistan

Higher Education Commission of Pakistan has been extending financial assistance to its recognized journals in various disciplines including health sciences to help overcome their financial difficulties which is indeed commendable. They have also laid down certain guidelines for evaluation and recognition of journals in different categories and they are regularly monitored and evaluated every year. More recently HEC has revised its publications policies regarding health science journals in its meeting held on August 30-31st 2019 which it has termed as a “major policy shift towards a more transparent, rigorous and focused system for Accountability, Monitoring Capacity Building and Funding of Research Journals”.^1^

While the objective is very commendable but the methodology HEC is adopting to realize these objectives, it is feared, might not achieve the desired results. On the contrary it may lead to the death of some of the health science journals thereby multiplying the problems which the researches in Pakistan are facing to get their research work published. Already we have only a few good quality standard peer reviewed biomedical journals and the waiting period for publication is quite long. At present only three medical journals in Pakistan have earned Impact Factor and they are under too much pressure from the authors who wish to get their manuscripts published as soon as possible. One thing with the authorities in HEC need to understand and realize is that being a distinguished research scientist, occupying a coveted post of Vice Chancellor of a medical university, Dean or Principal of a medical or dental college including those who somehow manage to find a place among the honorary editors of some journals published from overseas does not mean they are experts in the field of Journalology and they are capable of planning, guiding, advising, framing guidelines for the improvement of medical journals. Over the years Journalology has emerged as an important discipline in itself and this is an art which one has to learn for which on the job training is a must. Someone who has not actually edited or published a journal cannot understand the problems which the editors face and these problems are different in different parts of the world. These problems need indigenous solutions and one cannot transplant or implement any solution which works in the West in low income countries like Pakistan.^2^

Let us first look into the background before coming to the topic under discussion. While journal publishing has a very long history, it was in 1978 that International Committee of Medical Journals Editors (ICMJE) was founded and later World Association of Medical Editors got established.^3^,^4^ National Library of Medicine in USA established the Index Medicus and Medline became a popular indexing service. However, since the disease pattern and pathology in various countries was different, Medline was not interested in covering many of those issues which lead to the formation of different regional index Medicus by WHO. Since the problems which the editors were facing in different parts of the world were also different and it needed some indigenous, cost effective solutions, ICMJE and WAME was followed by emergence of various regional bodies of medical editors like Eastern Mediterranean Association of Medical Editors (EMAME), Asia Pacific Association of Medical Editors (APAME) and Federation of African Medical Editors (FAME).^5^-^7^ Then professional bodies of medical editors were formed in various countries and they are playing a vital role in helping, promoting the discipline of Journalology in China, Korea, Malaysia, India, Singapore, Vietnam, Magnolia, Iran, Japan, Philippines and other countries.^8^ Pakistan Medial Journalists Association which was renamed as Pakistan Association of Medical Editors (PAME) and got registered in 2010 has also played its role in promoting the discipline of medical journalism in Pakistan.^9^ Ever since its inception it has been busy in organizing workshops on scientific writing, peer review, publication ethics creating awareness about scientific misconduct and how to avoid that, training courses for medical editors with its limited resources.^10^ It regularly organizes its national conferences which are preceded with Hands on Workshops to build the professional capacity of editors. In collaboration with World Health Organization (WHO), College of Physicians & Surgeons of Pakistan, Aga Khan University and Dow University of Health Sciences, PAME also hosted the EMMJ5 Medical Journals Conference of EMAME held at CPSP Campus at Karachi in December 2010 which was attended by thirty four foreign delegates from eighteen countries besides three hundred delegates and participants from all over the country. The then Chairman of HEC Dr. Javed Leghari was the Chief Guest in the inaugural session. Proceedings of this conference were later published in a book form^11^ which is available in pdf format on PAME website. More recently in collaboration with University of Health Sciences Lahore, PAME has started a six months Certificate Course in Medical Editing^12^ which will be followed by an advance course in Medical Editing. The first batch of 29 doctors most of whom are highly qualified and have more than one postgraduate qualifications have completed the first basic course and their final assessment and results is expected in March 2020. Professional bodies of medical editors are helping the regulatory bodies in various countries to improve the standard of their biomedical journals.

Pakistan Journal of Medical Sciences has started an internship programme for junior editors which also carry a training scholarship of Rs. 25,000/- [$200] in memory of its late Founder Chief Editor Dr. Maqbool H.Jafary and so far six junior editors have been trained in scientific publishing.

Now let us look at some of the revised guidelines for the Health Science journals by the HEC in different categories. In the W category there are only three journals i.e. Pakistan Journal of Medical Sciences, Journal of Pakistan Medical Association and Journal of College of Physicians & Surgeons Pakistan. They all have proper set up with full time staff and they will somehow manage the requirements. HEC can also help and assist them to make up if there are any deficiencies. However, when we come to the X category journals, HEC requires that they should have one fourth of their papers with international authorship, no self-institutional Publications will be allowed and articles processing through OJS or similar journal management system is mandatory. Just imagine a journal which is not yet indexed in important world data basis, has no Impact Factor, how will it attract manuscripts with foreign authors. Similarly if the faculty members of the institution publishing the journal themselves do not publish their research work in their own journal, why others will be interested? The result will be such journals will eventually cease publication. What the HEC should have recommended that do not make it a House Journal; make sure that these journals also have some manuscripts from faculty members from other institutions while inviting manuscripts from overseas as well. These institutions should also be asked to help promote collaborative research with institutions overseas and then publish their research in their journals which will automatically ensure authors from overseas as well. The HEC should also have helped these journals to know how to use the Open Journal System first by arranging some training workshops, in fact HEC did arrange such course but since they do not have the expertise, it failed to make any worthwhile impact. The training courses it has organized also needs to be further improved to enhance their utility as one such course organized at Karachi on April 7, 2017 left much to be desired. In fact some of the eminent editors attending the course were much better informed than the facilitators which the HEC had arranged and they did point it out during the discussion.^13^

Before making it compulsory for the university faculty members to publish their papers in Impact Factor journals, HEC should have ensured that we do have enough journals with Impact Factor within the country. Publication in IF journals overseas costs between US$ eight hundred to two thousand at least and how many of our authors can afford it unless the research is sponsored and funded by some agency which is very rare. We had first convinced the Government of Pakistan about the usefulness and necessity of having a National Bioethics Committee which was eventually notified in 2003, then we helped establish provincial Ethics Committees, ECs in various medical institutions were also established and training workshops were organized for them for two years before the three leading IF journals of Pakistan made it compulsory for the authors to ensure that their submission is accompanied with Ethics Committee/Institutional Review Board approval. One has to create proper infrastructure before asking to comply with different guidelines.

For journals in Y Category HEC requires that the Editorial Board should have PhD degrees in relevant fields, area of publication and strong research and publication background. It will be interesting to find out how many such PhDs we have and how many of them are willing to contribute? Publications by Editor and members of the Editorial team are also not allowed. Moreover, it also mandates that self institutional authorship shall not exceed one fifth of the total articles which is of course acceptable. In cases, it will be extremely difficult for the newly started journals to comply with such requirements. While the objective of achieving some standardization and quality of health science journals is laudable but it is also important that regulatory bodies should act as facilitators and extend valuable help and assistance to the national journals to get indexed in important international databases, earn Impact Factor, increase their visibility by making their presence on the PubMed and PubMed Central. Instead of providing any financial grants, the HEC can organize hands on training workshops for Editors in scientific publishing, use of Open Journal System; provide software to prepare XML files for submission to PubMed Central which will increase the visibility of Pakistani journals and thus increasing our citation in world medical literature.^13^

There are well known and documented professional competencies which the editors of biomedical journals are supposed to be aware of and try to achieve them.^14^-^15^ PAME is trying its best to help its members to achieve these competencies as this will ensure the standard of their journals.

Pakistan Association of Medical Editors has the expertise. It has already organized three one day courses in the use of Open Journal System which the participants found quite useful. PAME intends to organize a two-day hands on workshop for Editors on OJS while it also has plans to organize similar workshops for the support staff of the editors in various journals because it is the support staff which are going to use the OJS system more frequently. The editors who are familiar with the OJS can then monitor their support staff more efficiently. Since most of the editors of journals being published by professional specialty organizations, medical institutions are part time, their first and foremost responsibility is teaching, training, research and patient care, they cannot devote much time for their journals. They need to have a team wherein this support staff will play a very vital role.

PAME has in the past offered its services to the regulatory bodies without any obligation and it is willing to help and assist them if they wish to improve the standard of biomedical journals being published from Pakistan. We need to bridge the communication gap which at presents exist between professional editors represented by PAME and the regulatory bodies. On its part, PAME will continue its humble contributions in this field as in the past with its limited resources because PAME members feel it is their duty and responsibility to train maximum number of people in the art of medical writing and scientific publishing.

## References

[1] HEC notification No. (22)/R&D(SS&H)/HEC/2019/337 dated November 5 (2019).

[2] Jawaid SA (2004). Problems faced by editors of peer reviewed medical journals. Saudi Med J.

[3] http://www.icmje.org/.

[4] https://www.wame.org/.

[5] Jawaid SA (2004). Birth of Eastern Mediterranean Association of Medical Editors (EMAME). Editorial. Pak J Med Sci.

[6] http://www.wpro.who.int/entity/apame/about/en/.

[7] https://www.who.int/tdr/publications/training-guideline-publications/fame-editorials/en/.

[8] Shaukat Ali Jawaid, Masood Jawaid (2017). Medical Writing:Basic Definitions and some useful information.

[9] https://www.pame.org.pk/.

[10] Jawaid SA (2016). Professionalism in Medical Journalism and Role of HEC, PM&DC. Annals of KEMU.

[11] https://www.pame.org.pk/Books/EMMJ-5_Proceedings/index.html.

[12] Jawaid SA, Jawaid M (2019). Professional capacity building of Health Science Journal Editors. Pak J Med Sci.

[13] Jawaid SA, Jawaid M (2017). What regulatory agencies like HEC, PM&DC can do to help improve quality and standard of Pakistani Biomedical Journals. Pak J Med Sci.

[14] Moher D, Galipeau J, Alam S, Barbour V, Bartolomeos K (2017). Core competencies for scientific editors of biomedical journals:consensus statement. BMC Medicine.

[15] Jawaid SA, Jawaid M (2017). Professional competencies required for Editors of Biomedical Journals. Pak J Med Sci.

